# Effectiveness of Manual Small-Incision Cataract Surgery in Treating White Cataracts

**DOI:** 10.7759/cureus.104136

**Published:** 2026-02-23

**Authors:** Riya Rani Kesh, Sasmita Pradhan, Rammohan G

**Affiliations:** 1 Ophthalmology, Sri Venkateshwaraa Medical College Hospital and Research Centre, Puducherry, IND; 2 Community Medicine, Srirama Chandra Bhanja Medical College, Cuttack, IND; 3 General Medicine, Sri Venkateshwaraa Medical College Hospital and Research Centre, Puducherry, IND

**Keywords:** anterior segment, cataract surgery, manual small incision cataract surgery, systemic comorbidities, visual outcome, white cataract

## Abstract

Aim: To evaluate the outcomes of manual small-incision cataract surgery (MSICS) in patients with white cataracts, including those with systemic comorbidities.

Introduction: Cataract remains a leading cause of blindness. MSICS is a cost-effective, safe technique suitable for white cataracts and patients with systemic conditions.

Materials and methods: This is a retrospective study from 108 patients (108 eyes) who underwent MSICS (May 2023 to October 2024). Preoperative assessments included ocular and systemic evaluations. Surgical outcomes and complications were analyzed.

Results: In the present study, 99% achieved best-corrected visual acuity (BCVA) ≥ 6/18, and 89% achieved ≥ 6/9 at 45 days. Intraoperative complications were rare (one case of posterior capsular rupture). Postoperative complications occurred in 18.5% of cases (corneal edema, iritis); all were resolved before discharge.

Conclusion: MSICS is safe, effective, and cost-efficient technique, offering excellent visual outcomes even in complex cases involving white cataracts and systemic comorbidities.

## Introduction

Cataract is the leading cause of treatable blindness worldwide, with a disproportionately higher burden in developing countries [1]. Delayed presentation and unmet cataract surgical needs driven by limited access to eye care services, socioeconomic constraints, and healthcare disruptions, including those observed during public health crises such as the COVID-19 period have contributed to the progression of cataracts to advanced or white stages in many patients [1-3]. The World Health Organization (WHO) estimates that, as of 2021, approximately 2.2 billion people globally experience near or distance vision impairment. Among these, more than 1 billion individuals have moderate to severe distance vision impairment, and nearly 94 million cases are attributable to cataract [2]. Cataract alone accounted for approximately 45% of global blindness in 2020 and represents the second most common cause of moderate to severe vision impairment (MSVI) worldwide [2-5].

The substantial global burden of cataract-related visual impairment highlights the critical role of timely and effective surgical intervention in restoring vision and improving quality of life. Cataract-induced visual disability significantly affects individuals’ social independence, productivity, and overall well-being, thereby contributing to broader socioeconomic consequences, including increased disability and premature mortality [6].

The classification of white cataracts is based on the level of lens maturation and includes incipient, immature, mature, hypermature and intumescent cataracts. A mature cataract is a condition in which the crystalline lens is completely opaque, resulting in severe visual impairment, and a hypermature cataract is an advanced stage in which the cortical fibers are liquefied, the capsular integrity is compromised, and intraoperative complications are likely to occur. Intumescent cataracts, which are commonly classified as part of white cataracts, are associated with swelling of the lens and increased intralenticular pressure, making capsulorhexis difficult to perform. Population-based studies in India have indicated that the burden of advanced and operable cataracts among the aging population is high, which is mainly due to unmet surgical needs, socioeconomic factors, and limited access to healthcare services [1,2]. 

Cataract surgery remains the most effective treatment modality, offering significant improvements in visual acuity and daily functioning while reducing cataract-associated morbidity and mortality [1]. Among the various surgical techniques available, manual small-incision cataract surgery (MSICS) is widely practiced in resource-limited settings and has been demonstrated to be a safe, cost-effective, and efficient method for cataract extraction [7]. Unlike phacoemulsification, MSICS does not rely heavily on advanced technology or expensive consumables, making it particularly suitable for high-volume surgical settings. Additionally, MSICS is associated with shorter operative times, reduced dependence on ultrasound energy, and comparable visual outcomes, especially in advanced cataracts. However, the technique requires surgical expertise and careful wound construction to minimize surgically induced astigmatism and intraoperative complications.

Surgical management of advanced cataracts, including mature, hypermature, and intumescent cataracts, poses unique challenges due to factors such as poor red reflex, increased intralenticular pressure, and compromised capsular integrity. Systemic comorbidities, such as diabetes mellitus, hypertension, and cardiovascular disease, are common in the elderly population and may complicate preoperative medical fitness for surgery.

The grade or morphological type of cataract itself does not have a direct causal relationship with these systemic conditions. Nonetheless, optimizing surgical technique and perioperative management is essential to achieve favorable outcomes in this patient population.

The objective of the present study was to assess visual outcomes and perioperative complications following MSICS in patients with white cataracts, including those with systemic comorbidities.

## Materials and methods

Study design and patient selection

This retrospective study was conducted in the Department of Ophthalmology at a tertiary care center in Puducherry after obtaining institutional ethical clearance (Ref No: 57/SVMCH/IEC-Cert/June-25). The study included 108 eyes of 108 consecutive patients diagnosed with mature, hypermature, or intumescent cataracts who underwent MSICS by a single surgeon between May 2023 and October 2024. Patients with congenital or traumatic cataracts, glaucoma, uveitis, previous intraocular surgery, or pre-existing corneal pathology were excluded.

Patient enrollment and preoperative assessment

All patients were enrolled through outreach camps conducted by the institution and underwent surgery free of cost. Preoperative assessment included detailed medical history, general, ocular, and systemic examination, along with routine blood and urine investigations. Patients with abnormal systemic parameters were referred to the appropriate specialty departments, and surgery was undertaken only after medical clearance. Patients with cardiac disease or hypertension were operated under anesthetist's supervision.

Management of systemic comorbidities

Diabetes mellitus was initially screened using random blood sugar and urine sugar testing. If positive, fasting blood sugar (FBS) and postprandial blood sugar (PPBS) were assessed. Patients were referred for medical management if FBS ≥126 mg/dL or PPBS ≥200 mg/dL, and surgery was scheduled only after achieving FBS ≤140 mg/dL and PPBS ≤200 mg/dL. Hypertensive patients underwent surgery only after adequate blood pressure control.

Ophthalmic evaluation and cataract classification

All patients underwent detailed anterior and posterior segment evaluation. Each patient was screened for a patent nasolacrimal duct and a negative regurgitation test. Pupillary dilation was achieved using tropicamide 0.8% with phenylephrine 5%, except in patients with hypertension or cardiac disease, in whom tropicamide 1% alone was used.

Cataracts were classified based on standard clinical morphological descriptions commonly used in cataract surgery practice, considering anterior chamber depth, appearance of the anterior capsule, and consistency of lens matter.

Mature cataracts were defined by completely opaque lens matter with normal anterior chamber depth. Hypermature cataracts were characterized by liquefied milky cortex with fibrotic anterior capsule. Intumescent cataracts were identified by swollen, hydrated lens matter associated with shallow anterior chamber depth.

Biometry and surgical preparation

Intraocular pressure was measured using a non-contact tonometer. Keratometry and A-scan biometry were performed using an Appasamy A-scan biometer, with the contact technique in all patients. Intraocular lens (IOL) power was calculated using the Sanders-Retzlaff-Kraff II (SRK-II) formula [8,9]. Posterior segment pathology was ruled out in all cases using B-scan ultrasonography.

Topical ciprofloxacin 0.3% eye drops were started two days prior to surgery. On the day of surgery, the operative eye was prepared with 5% povidone-iodine, and peribulbar anesthesia was administered after achieving adequate mydriasis.

Surgical technique

All procedures were performed under strict aseptic precautions using standard MSICS technique. A superior fornix-based conjunctival flap was created, followed by a 6-6.5 mm frown-shaped scleral incision and scleral tunnel construction. Trypan blue-assisted capsulorhexis was performed in all cases. The nucleus was delivered using an irrigating vectis, and cortical matter was aspirated using a Simcoe cannula. A single-piece polymethyl methacrylate (PMMA) IOL was implanted in the capsular bag.

The scleral tunnel and conjunctival flap were closed without sutures. Viscoelastic was completely removed, and balanced salt solution was used to reform the anterior chamber. Intraoperative complications, if any, were documented.

Postoperative care

Postoperative evaluation was performed on day one in all patients. Standard postoperative treatment included topical ciprofloxacin 0.3% and dexamethasone 0.1% eye drops. In diabetic patients, topical flurbiprofen 0.03% was prescribed in addition to steroid drops for four weeks.

Classification of complications

Intraoperative and postoperative complications were graded using the Oxford Cataract Treatment and Evaluation Team (OCTET) classification [10].

Follow-up schedule

Patients were reviewed on postoperative days 1, 7, 30, and 45. Uncorrected and best-corrected visual acuity (BCVA) were recorded at each visit. A comprehensive ophthalmic examination, including slit-lamp evaluation, fundoscopy, and refraction, was performed at the final follow-up.

Statistical analysis

Data were entered into Microsoft Excel and analyzed using Statistical Package for the Social Sciences (SPSS) version 23. Categorical variables were expressed as frequencies and percentages, and continuous variables as mean and standard deviation (SD). Inferential statistical outcomes are presented in the Results section.

## Results

Preoperative data

**Figure 1:**
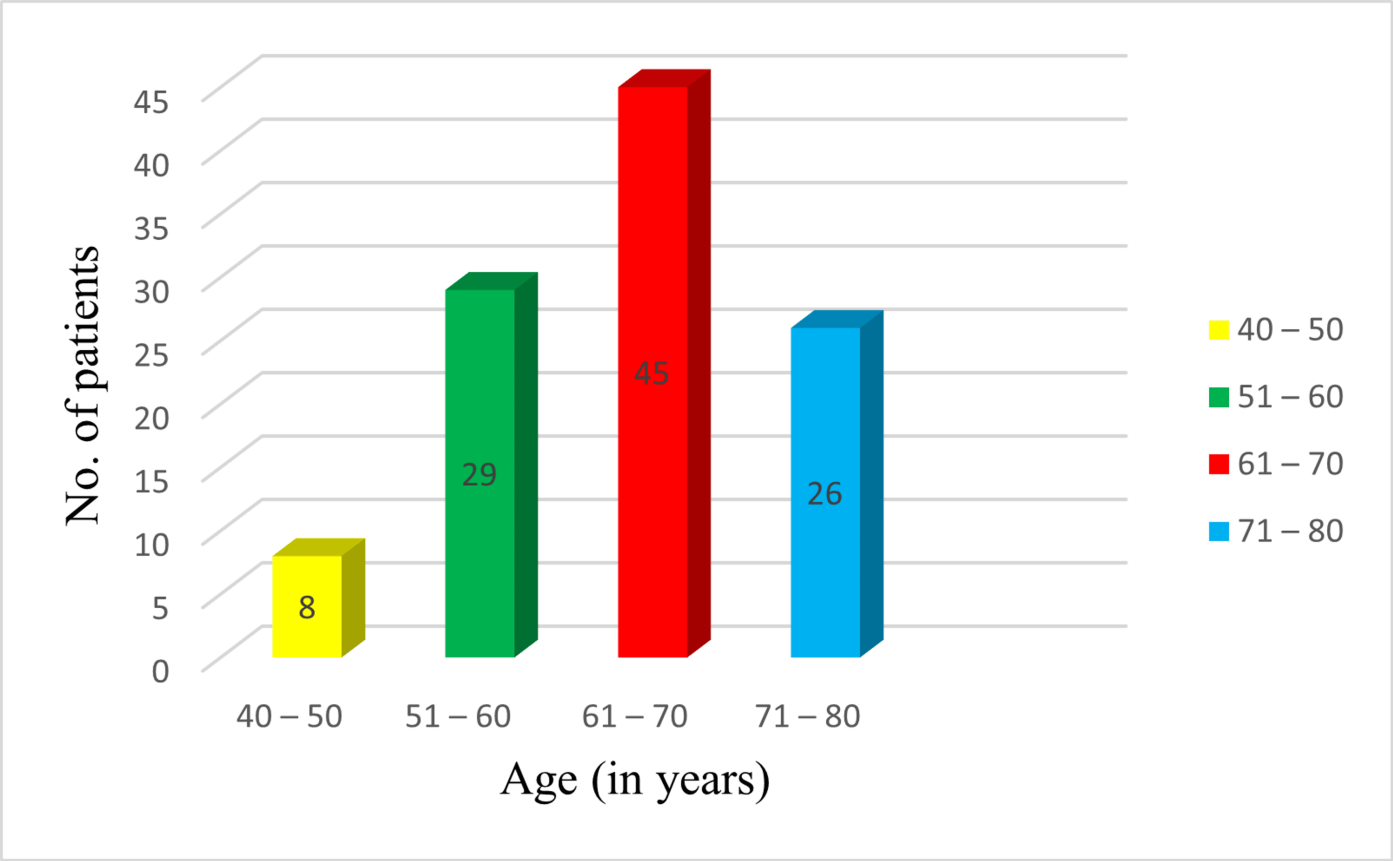
Distribution of study population according to age

The study included a diverse cohort of patients across various age groups. The majority of cases were observed in the age group of 61-70 years, accounting for 45 cases (41.67%), followed by 29 cases (26.85%) in the age group of 51-60 years, 26 cases (24.07%) in the age group of 71-80 years, and eight cases (7.41%) in the age group of 40-50 years (Figure 1). 

Regarding the lens status in the non-operated eyes, 48 eyes (44.44%) were pseudophakic, 52 eyes (48.15%) had immature cataracts, seven eyes (6.48%) had mature cataracts, and one eye (0.93%) had a hypermature cataract (Table 1).

**Table 1:** Characteristics of the study population (N=108) Visual acuity measured using the Snellen chart (public-domain tool) [13]. Cataract maturity classification follows descriptions from Venkatesh et al. [14]. BCVA: Best-corrected visual acuity; FCF: Finger counting close to face; HM: Hand movements; PL: Perception of light

Parameters	No. of patients (%)
Gender	
Male	41 (38)
Female	67 (62)
Laterality of the operated eye	
Right	62 (57.4)
Left	46 (42.6)
Preoperative visual acuity in the operated eye (BCVA)	
FCF	2 (1.85)
HM	56 (51.85)
PL	50 (46.3)
Lens status in the non-operated eye	
Pseudophakia	48 (44.44)
Immature cataract	52 (48.15)
Mature cataract	07 (6.48)
Hypermature cataract	01 (0.93)

Female patients constituted a higher proportion of the study population, accounting for 67 cases (62%), compared to 41 male patients (38%).

**Figure 2:**
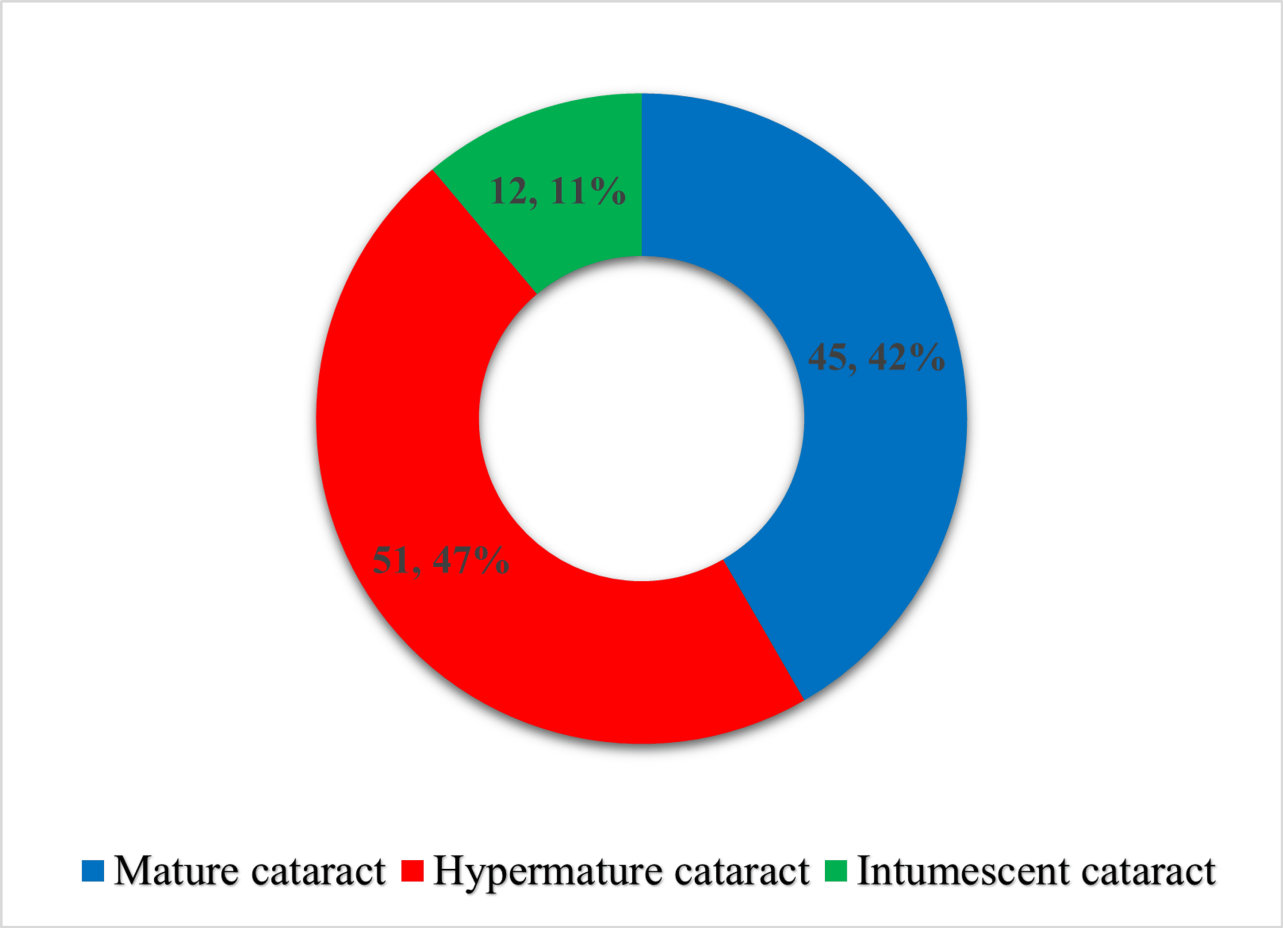
Distribution of subjects according to the type of cataract in the operated eye Cataract maturity definitions are based on established clinical criteria [14,15].

Preoperatively, visual acuity varied significantly among the patients. Two eyes (1.85%) had a vision of finger counting close to face (FCF), 56 eyes (51.85%) had hand movements (HM), and 50 eyes (46.3%) had perception of light (PL) (Table 1). The cataracts were categorized into three types: 45 cases (41.67%) of mature cataracts, 51 cases (47.22%) of hypermature cataracts, and 12 cases (11.11%) of intumescent cataracts (Figure 2).

**Figure 3:**
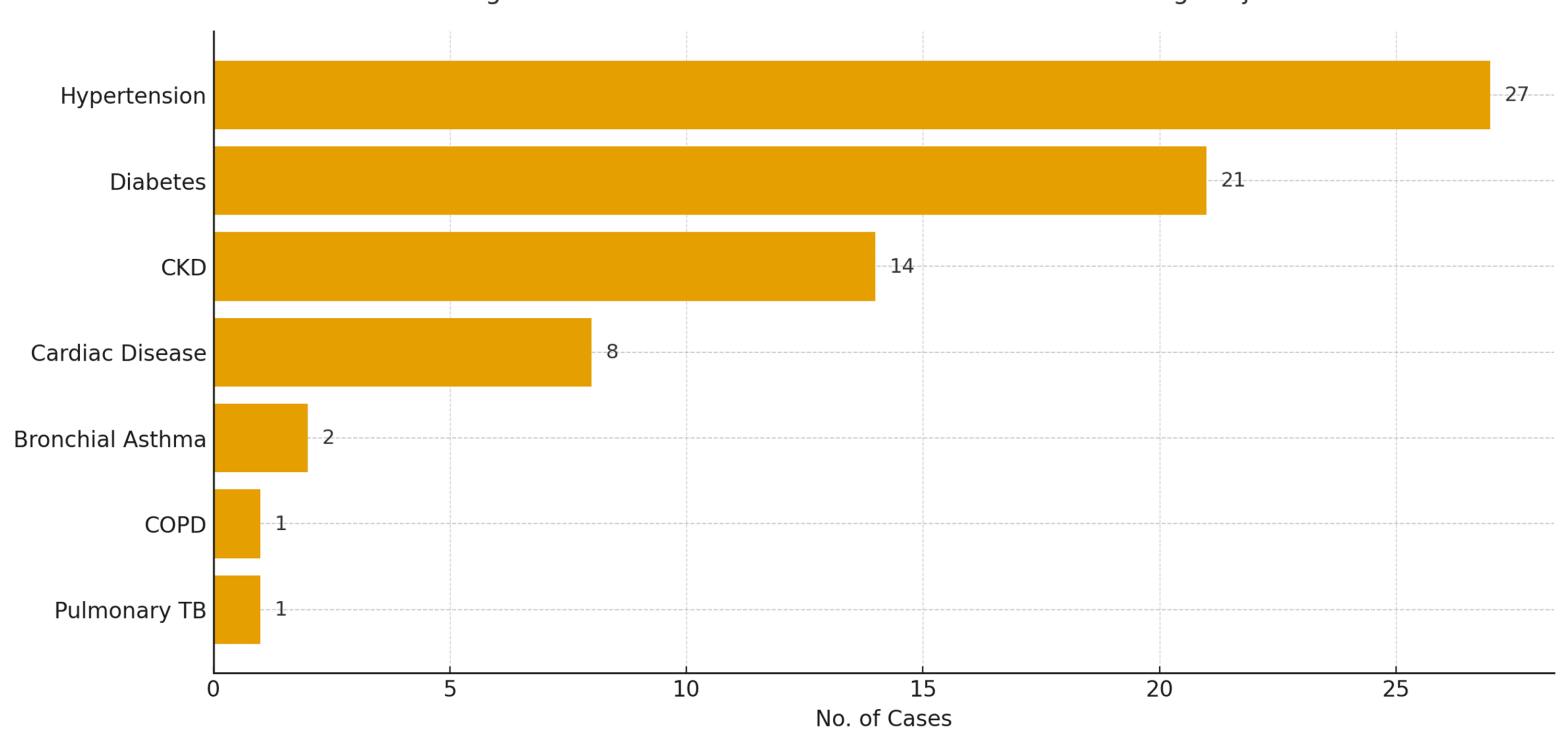
Distribution of associated comorbidities among subjects

Several systemic comorbidities were observed among 74 cases (68.52%). Hypertension was present in 27 cases (25%), followed by diabetes mellitus in 21 cases (19.4%), chronic kidney disease (CKD) in 14 cases (13%), and cardiac disease in eight cases (7.4%). Other comorbidities included bronchial asthma in two cases (1.9%), chronic obstructive pulmonary disease (COPD) in one case (0.9%), and a past history of pulmonary tuberculosis (treated and inactive) in one case (0.9%) (Figure 3).

The preoperative biometric measurements were recorded with mean values and SDs as follows: flat keratometry readings (K1) averaged 45.05 ± 1.82 D, steep keratometry readings (K2) averaged 45.37 ± 1.63 D, axial length averaged 22.55 ± 0.80 mm, and the mean IOL power was 21.32 ± 1.82 D (Table 2).

**Table 2:** Mean and SD of K1, K2, axial length and IOL power in the operated eyes IOL power calculated using the SRK-II formula [8,9,16]. K1: Flat keratometry readings; K2: Steep keratometry readings; IOL: Intraocular lens; SRK: Sanders-Retzlaff-Kraff

Variable	Mean ± SD
K1	45.05±1.82
K2	45.37±1.63
Axial Length	22.55±0.80
IOL Power	21.32±1.82

Intraoperative data

Out of the 108 eyes operated, 62 (57.4%) were in the right eye and 46 (42.6%) were in the left eye. Capsulorrhexis extension was observed in one intumescent cataract, then the capsulotomy was completed using Utratacapsulorrhexis forceps from the opposite direction. In the remaining 107 eyes, continuous curvilinear capsulorhexis (CCC) was completed successfully. One eye had posterior capsular rupture without vitreous loss and three-piece IOL was placed in the sulcus. None of the eyes had zonular dialysis or iridodialysis.

Postoperative data

On the first postoperative day, complications were observed in a subset of patients. According to OCTET classification, corneal edema with Descemet’s folds < 10 was noted in nine eyes (8.33%), corneal edema with Descemet’s folds > 10 developed in five (4.63%) eyes.

Mild iritis was observed in four eyes (3.7%), while moderate iritis with fibrin membrane was seen in two eyes (1.85%). Of the 108 eyes operated, 107 eyes had posterior chamber IOL (PCIOL) in the bag and one eye had three piece IOL in the sulcus. The overall postoperative complication rate was 18.5%, encompassing complications of varying degree (Table 3). All postoperative complications were effectively managed, and resolved before the patients were discharged. Patients without any postoperative complications were discharged on the second day after surgery, whereas those with moderate iritis were discharged on the third postoperative day. For postoperative uveitis, homatropine 2% eye drops every 6hours was added along with standard regimen.

**Table 3:** Findings on postoperative day 1 (N=108) Complications classified using the OCTET system [10]. OCTET: Oxford Cataract Treatment and Evaluation Team

Postoperative findings (OCTET classification)	No. of patients (%)
Corneal edema with Descemet’s folds < 10	9 (8.33)
Corneal edema with Descemet’s folds > 10	5 (4.63)
Mild iritis < 50 cells	4 (3.7)
Moderate iritis > 50 cells with fibrin membrane	2 (1.85)
Total	20 (18.5)

**Figure 4:**
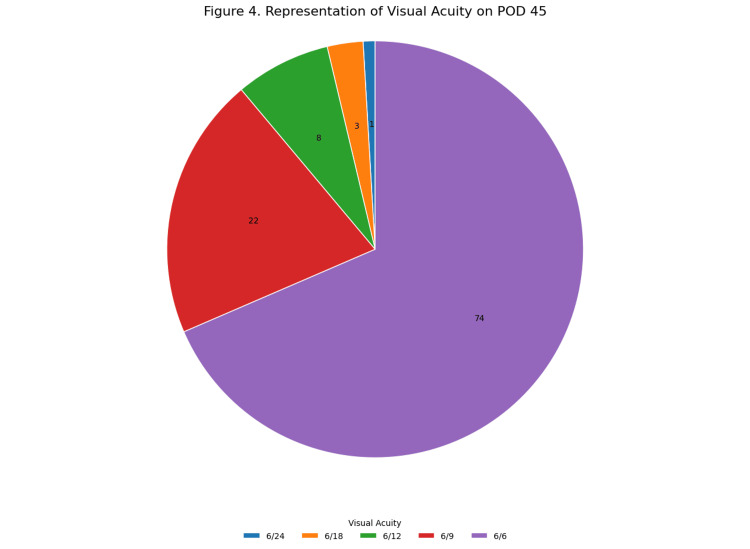
Representation of visual acuity on postoperative day 45 Postoperative visual acuity outcomes following WHO (1998) cataract outcome recommendations [17]. WHO: World Health Organization

On the 45th postoperative day, 107 patients (99%) achieved BCVA of 6/18 or better. Among these, 89% of patients achieved BCVA of 6/9 or better (Table 4). Categorizing the postoperative visual acuity according to the World Health Organization (WHO) guidelines - good outcomes (6/6 to 6/18), borderline outcomes (<6/18 to 6/60), and poor outcomes (<6/60), none of the patients fell into the poor outcomes category (Figure 4).

**Table 4:** Postoperative visual acuity (by Snellen’s chart) (N=108) Outcome classification is based on WHO cataract surgery outcome guidelines [17]. BCVA: Best-corrected visual acuity; WHO: World Health Organization

BCVA	No. of eyes			
	Postoperative day 1	Postoperative day 7	Postoperative day 30	Postoperative day 45
3/60	-	-	-	-
6/60	3	3	-	-
6/36	9	6	2	-
6/24	21	22	13	1
6/18	27	23	17	3
6/12	22	15	29	8
6/9	19	23	23	22
6/6	7	16	24	74

## Discussion

This study examined the results of MSICS in eyes with white cataracts, many of which were from older patients with multiple systemic comorbidities. These results must be seen in the context of the enormous global burden of cataract. Evidence from India shows that while the unmet need for cataract surgery in the ageing population has started to decline, a significant number of older people still live with operable cataract related visual impairment [1]. At the global level, WHO and the Global Burden of Disease estimates confirm that cataract remains a leading cause of blindness and vision impairment worldwide, particularly in low- and middle-income regions [2-4]. Socioeconomic analyses further demonstrate that cataract surgical rates are closely linked to national income and health system capacity, with lower rates in poorer regions [5]. Together, these findings emphasize the ongoing demand for effective, scalable surgical approaches like MSICS in regions with a high burden of cataract.

The provision of cataract surgery in low-resource countries poses unique challenges: Patients usually arrive late with dense and white cataracts and limited access to regular eye care, and health systems are forced to manage large volumes of surgeries on limited infrastructure [6]. MSICS has been proposed as a suitable alternative in such situations, as it uses less specialized equipment and is less expensive than phacoemulsification, but can achieve similar visual results as phacoemulsification when done by trained surgeons [6,7]. This is supported by our research: Even in the case of advanced white cataracts, we obtained a high percentage of patients with good visual acuity postoperatively and a low rate of severe complications.

Good refractive outcomes require accurate biometry and IOL power calculation. In our case series, we used a combination of keratometry and axial length measurements with the SRK II formula to choose the correct IOL power. The SRK II, as defined by Sanders et al. and further explained by Holladay, Dang and Raj, is a popular regression-based technique in clinical practice, particularly when the newer-generation formulae or optical biometers are not universally accessible [8,9,16]. The small range of postoperative visual acuity and the large percentage of eyes with BCVA of 6/9 or higher suggests that, with the aid of good biometry, SRK II remains effective in standard cataract surgery, even in difficult white cataracts.

The grading of complications was based on the OCTET system, which is a validated system that is regularly applied in trials and audits [10]. This method facilitated the uniform categorization of the early postoperative events, such as corneal edema and iritis. The total complication rate was 18.5%, which was mainly mild and temporary alterations of the anterior segment. This number is similar to and in some ways superior to those found in tertiary centers in sub-Saharan Africa, where patients often present with severe visual impairment and other ocular comorbidities [11,12]. The percentage of eyes with hand-motion vision or worse at presentation was large in those series [11,12]. In our cohort, most eyes were also severely visually impaired at the time of surgery, with visual acuity measured using the standard Snellen chart [13].

Our visual results were equal to or better than those of other MSICS series for white or dense cataracts. Venkatesh et al. demonstrated that MSICS can provide good outcomes in white cataract eyes with high rates of good postoperative visual outcomes and a moderate complication profile [14]. Similarly, Singh et al. found that MSICS is a flexible technique that can be applied to a broad spectrum of cataract densities and clinical environments, such as high-volume programmes in developing nations [15]. Our series had 99% of eyes with a BCVA of 6/18 or higher, and 89% with 6/9 or higher by day 45 - values that are very favorable compared to these previous reports and that MSICS is a safe method in challenging cataract cases.

We have found that in a group with a high prevalence of systemic comorbidities, such as hypertension, diabetes, and CKD, which is typically seen among cataract patients in most low- and middle-income populations, we have obtained our results [6,11,12]. These comorbidities did not contribute to a higher rate of intraoperative complications in our series after proper preoperative optimization and anesthetic support. This shows that MSICS can be safely administered to patients with multiple comorbidities when there is multidisciplinary care and perioperative monitoring.

The strengths of this study include a standardized surgical technique performed by a single surgeon, validated biometry and IOL calculation procedures, and the use of internationally recognized frameworks to grade complications and evaluate outcomes [10,17]. The visual outcomes were measured by using WHO cataract surgery outcome categories, which established a standard that 80% of the eyes should have a postoperative vision of 6/18 or higher [17]. Our results were much higher than this, demonstrating the high quality and efficiency of MSICS.

This study is limited by its retrospective design, single-center setting, and relatively short follow-up period, which does not capture long-term refractive stability or late complications such as posterior capsular opacification. Additionally, no comparison group undergoing conventional phacoemulsification was included. Despite these limitations, our results strongly recommend MSICS as a safe, efficient, and effective method of treating white cataracts in regions where cataracts are major cause of preventable blindness.

The study also highlights the feasibility of performing MSICS in patients with systemic comorbidities, such as hypertension, diabetes, and cardiac conditions. These patients were managed with appropriate preoperative optimization and intraoperative monitoring, resulting in favorable outcomes.

## Conclusions

MSICS is a safe, cost-effective, and reliable method of managing cataracts, with good visual results even in complicated cases, including patients with systemic comorbidities. This study highlights the significance of MSICS as a viable and sustainable alternative to cataract surgery, especially in areas that have a low healthcare infrastructure and access to modern surgical devices. The results justify the continued application of MSICS as a useful and effective method of attaining the best visual recovery in a wide range of patients.
